# Joint Maximum Likelihood Time Delay Estimation of Unknown Event-Related Potential Signals for EEG Sensor Signal Quality Enhancement

**DOI:** 10.3390/s16060891

**Published:** 2016-06-16

**Authors:** Kyungsoo Kim, Sung-Ho Lim, Jaeseok Lee, Won-Seok Kang, Cheil Moon, Ji-Woong Choi

**Affiliations:** 1 Department of Information & Communication Engineering, Daegu Gyeongbuk Institute of Science and Technology, Daegu 771-813, Korea; ssi09@dgist.ac.kr (K.K.); ishdgist@dgist.ac.kr (S.-H.L.); jayslee@dgist.ac.kr (J.L.); 2 Wellness Convergence Research Center, Daegu Gyeongbuk Institute of Science and Technology (DGIST), Daegu 771-813, Korea; wskang@dgist.ac.kr; 3 Department of Brain & Cognitive Sciences, Daegu Gyeongbuk Institute of Science and Technology (DGIST), Daegu 771-813, Korea; cmoon@dgist.ac.kr

**Keywords:** EEG, ERP, maximum likelihood (ML), time delay estimation (TDE), synchronization

## Abstract

Electroencephalograms (EEGs) measure a brain signal that contains abundant information about the human brain function and health. For this reason, recent clinical brain research and brain computer interface (BCI) studies use EEG signals in many applications. Due to the significant noise in EEG traces, signal processing to enhance the signal to noise power ratio (SNR) is necessary for EEG analysis, especially for non-invasive EEG. A typical method to improve the SNR is averaging many trials of event related potential (ERP) signal that represents a brain’s response to a particular stimulus or a task. The averaging, however, is very sensitive to variable delays. In this study, we propose two time delay estimation (TDE) schemes based on a joint maximum likelihood (ML) criterion to compensate the uncertain delays which may be different in each trial. We evaluate the performance for different types of signals such as random, deterministic, and real EEG signals. The results show that the proposed schemes provide better performance than other conventional schemes employing averaged signal as a reference, e.g., up to 4 dB gain at the expected delay error of 10°.

## 1. Introduction

Electroencephalograms (EEGs) measure electrical brain activity using various EEG sensors, *i.e.*, dry type electrodes [1,2] and hydrogel based electrodes [3]. EEG data contains functional brain information, so EEG analysis has recently been utilized for many applications such as brain computer interface (BCI) [4,5,6], human cognition studies [7,8,9], diagnosis of diseases such as seizures and epilepsy detection [10,11,12]. EEG signals usually contain significant noise caused by background brain activity and system noise. This is far more significant in non-invasive interface EEG measurements. Therefore, signal processing to enhance the signal to noise power ratio (SNR) is necessary for EEG analysis. A typical method to enhance the SNR is by measuring many trials of event-related potential (ERP) signals that represent a brain’s response to a particular stimulus or a task and then averaging the trials to obtain ERP data with less noise. Averaging trials reduces noise power, thus enabling enhancement of the ERP signal SNR. This is based on the fact that ERP has the same signal waveform in the trials if there is no delay error in them, but the background brain activities that are not related to the event are independent in each of the trials. However, the averaging does not always enhance the SNR when the time delay in trials is uncertain. It has to be correctly estimated and shifted to correct the misalignment that is caused by variable time delays in the measured signal. This is because of the biological nature of the underlying processes or systematic measurement system timing errors. That is, even though many trials are averaged, the result does not show clear ERP features such as P3 under time uncertainty conditions [13]. The uncertain time delay has been reported as latency jitter. Recently, it is noticed that the ERP waveform and signal quality are influenced by latency jitter [14,15], and an EEG analysis method which applies a realigning method to average the trials with reduced time latency jitter has been studied for better results [16]. These studies report that the latency jitter is not only one of observations to understand brain responses to stimuli but also a problem that contaminates the averaged ERP signal. This paper considers this latency jitter as a time delay error of the ERP signal in each trial and proposes algorithms that can obtain more accurate latency jitter and compensate the time delay to enhance EEG signal quality.

The time delay estimation (TDE) technique involves estimating variable time delays in multiple signal sources. TDE has been studied not only in the biomedical engineering field, but also other fields such as sound signal processing. In sound signal processing the TDE method tries to estimate receiving sound delays from multiple microphones (Mics). The TDE result can provide relative distance between multiple Mics and the sound source. In [17] cross-correlation based TDE algorithm was applied, and in [18] a mutual information based algorithm was applied to localize and track a speech signal. Note that, since the relative distance of Mics is known for TDE in sound signal processing, the time delays of multiple Mics are linked, thus the TDE error can be compensated.

Several studies have been conducted for TDE in the biomedical engineering field. Note that in this case the situation is different from TDE for sound signal processing since the time delays in repetitive biomedical signal are independent, while TDE in sound signal processing assumes that the delays are relative due to the relative distance between multiple Mics. The TDE studies in the biomedical field focus on searching for each independent time delay based on the estimation of a common signal waveform that is called a template. Woody’s method [19] iteratively calculates the cross-correlation between each trial signal and a template or a reference signal where the template signal is obtained by averaging the trial signals whose delay is shifted by the delay estimated in the previous iteration. Based on the template estimation, the authors of [19] estimate each single trial time delay individually. Pham *et al.* [20] proposed a variable delay estimation method based on maximum likelihood (ML) in the frequency domain where an iterative Fisher-scoring method is applied to solve the ML problem to estimate the phase shift that is equivalent to the time delay in a time domain. These schemes can search the time delays for many trials quickly in an iterative way. Since ERP differs for people and is unclear, especially for signals measured from non-invasive interfaces [21,22], it is hard to know the ERP signal information in advance. Thus, these two schemes [19,20] estimate the ERP signal waveform to compute the time delay instead of taking prior information such as ERP signal information. In [19] the template is calculated, which is the same as ERP signal estimation, at each iteration by averaging each trial, and in [20] the signal spectrum is estimated by averaging trial spectra and spectrum variance. In addition, [23] presented a method, named the improved Woody scheme, that calculates the template more frequently than Woody’s method by averaging each trial after every cross correlation execution. 

These three techniques [19,20,23] use an iterative method to estimate variable time delays in trials individually. However, the templates in [19,23], and the estimated signal spectrum in [20] may cause TDE performance degradation. This is because the time delay estimation and the estimation of other parameters such as the template estimation are intertwined, and thus time delay estimation performance is affected by the estimation accuracy of the other parameters. This means that if the ERP signal is known, these conventional schemes provide the optimum solution, but without a perfectly known ERP signal, *i.e.*, if an accurate template is not available, they cannot avoid performance degradation. In addition, these conventional schemes estimate the time delay sequentially. At each step, they estimate a single delay for a trial signal, and then they sequentially estimate all the individual delays separately. The delays for all the trials are applied to generate the new template for the next iteration. Hence, the conventional TDE cannot estimate the time delay for all the trial signals simultaneously since each trial delay is estimated sequentially, using the template estimated at the previous iteration. Even though they iteratively estimate the time delay until the estimated delays converge, the performance may not reach the optimum solution. On the other hand, there is another study that simultaneously estimates time delays using a multiple correlation method [24]. Since the multi-trial time delays are estimated at the same time, the multiple correlation method [24] does not require ERP signal information as a template. However, since the method is derived in an intuitive manner, there may be performance degradation compared to the optimum scheme.

In order to prevent the performance degradation due to these problems, we propose two TDE schemes based on a joint ML criterion that estimates time delays of all the trial signals simultaneously without ERP signal estimation, applying a time delay set that is a vector composed of all time delays. In this paper, we first derive an optimum joint ML solution based on the joint probability density function (pdf) of the entire discrete-time signal. The optimum joint ML estimation, however, has much higher complexity than the iterative single delay estimation in [19,20,23] since it requires large scale matrix operation whose computation load is proportional to the data size and the number of possible candidates for the delay vectors, which increases exponentially as the number of trials increases. Thus, we also propose a lower-complexity sub-optimum joint ML scheme by taking an approximation of the most complicated operation in the optimum joint ML scheme. Although the sub-optimum scheme experiences a slight performance degradation over the optimum joint ML scheme, it can be implemented with significantly less complexity.

In short, these proposed schemes can provide optimum or near optimum performance without estimating an ERP or a template signal. Since the proposed TDE schemes perform in a joint manner that estimates all-trial delays simultaneously, they do not need additional parameter estimation such as the template to estimate one single trial delay based on the other trial delays. The optimum TDE requires only an autocorrelation function of the ERP signal which contains the ERP signal covariance information in multiple trials instead of the ERP signal itself, and the sub-optimum TDE can be applied without any ERP signal information such as mean and variance. They can obtain this by estimating delay vectors for all trials instead of a single trial time delay estimation. The previous studies [19,20,23] estimated single trial delays in a sequence, while our proposed TDE schemes estimate the delay vector composed of all-trial time delays that provides the best relative trial delays from a joint ML point of view, enabling better performance. 

This paper addresses the proposed schemes in the following steps: Section 2 introduces the signal model and conventional TDE schemes, while Section 3 describes the proposed optimum and sub-optimum TDE schemes. The performance is compared in Section 4 through computer simulation for various signal waveforms and ERP analysis. Finally, conclusions are drawn in Section 5.

## 2. Signal Model and Conventional TDE Schemes

A measured electrical EEG signal at discrete-time sample *n* = [1, 2, 3,…, *N*] of the *i-*th trial *i* = [1, 2, 3,…, *I*] can be represented as:

(1) $$y_{i}(n)=x(n-d_{i})+z_{i}(n)$$

where *x*(*n*) is an ERP signal having an average power of $\left(\mu_{x}^{2}+\sigma_{x}^{2}\right)$ with mean *μ_x_* and variance $\sigma_{x}^{2}$, *d_i_* is the time delay of the *i-*th trial, and *z_i_*(*n*) is noise such as background brain activities; this is assumed to be zero mean white Gaussian noise with variance $\sigma_{z}^{2}$ [19,20]. In order to generalize the problem, we treat signals as real value variables in this paper since an EEG signal is measured in real values.

The previous studies [19,23] estimate the time delay based on this model. They first estimate the ERP signal *x*(*n*), called template *p*(*n*), and utilize the cross-correlation between the template *p*(*n*) and the trials. In those studies, the time delay for the *i-*th trial signal is estimated by searching for the delay that has the maximum correlation coefficient, as:

(2) $${\hat{d}}_{i}^{q}=\underset{{\bar{d}}_{i}^{q}}{\arg\;\max}\frac{1}{N}\sum_{n=1}^{N}y_{i}(n+{\bar{d}}_{i}^{q})p^{q}(n)$$

where ${\hat{d}}_{i}^{q}$ is the estimated delay of the *i-*th trial signal at the *q-*th iteration. The template, which represents the averaged trials as $p^{q}(n)=\frac{1}{I}\sum_{i=1}^{I}y_{i}(n+{\hat{d}}_{i}^{q-1})$, gets closer to the ERP signal hidden in the measured signal, *i.e.*, *p*(*n*) ≈ *x*(*n*), by performing this process iteratively until the estimated delays converge. It should be noted that these schemes search for the solution in an iterative way. Their computation is sufficiently fast, even if there are many trials, *i.e.*, the Woody scheme [19] updates the template with the estimated time delay once in each iteration. As shown in Equation (2), only a single time delay ${\hat{d}}_{i}^{q}$ can be estimated by a single cross-correlation calculation. Therefore, the delays for all-trial are estimated step by step individually, and the delay estimation process is done at the end of an iteration. It should be noted that the estimated all-trial delays ${\hat{d}}_{i}^{q}$, *i* = 1, 2, 3,…, *I*, are updated to renew the template at the end of iteration *q*, so that the template estimation, which is the ERP signal estimation, is affected by the estimated delays in the previous iteration (*q* − 1). Hence, the template estimation and single delay estimation are intertwined. The improved Woody scheme [23] updates the template more frequently after each correlation operation, *i.e.*, every *i* and *q*, and this can make the template depend on the current time delays of the other trials that have been already estimated. However, the improved Woody scheme still has this intertwined problem since the *i-*th cross correlation in an iteration uses its template updated with the (*i* − 1) delays currently estimated.

Even TDE methods in the spectrum domain experience the same problem. The authors of [20,25] estimate the signal spectrum in a frequency domain by averaging the signal spectrum of each trial signal, however the time domain representation of the averaged signal spectrum yields the same result as the time domain averaging, which is the same as the template estimation. Thus, these methods suffer from the same problem because they have to perform signal spectrum estimation and delay estimation recursively for each trial.

According to these previous studies, the other variable estimation such as ERP signal estimation cannot be separated from TDE. Hence, estimation of time delay and of the template, treated as the reference signal, are intertwined and thus time delay estimation performance is affected by the accuracy of the template estimation. In addition, they estimate the delay of each trial individually without considering other delays ${\hat{d}}_{i}^{q}$, *i* ≠ *j*. On the other hand, there is another study that estimates time delay simultaneously by applying the multiple correlation method [24]. The correlation matrix can be reformed by multiple trial delays applied simultaneously, and the summation of the elements in the correlation matrix is calculated to search for the maximum correlation delay set, enabling TDE without additional parameter estimation. However, the algorithm has performance limitations since it is not derived from a statistical and mathematical approach. In addition, the performance may be further degraded using a differential evolution method which can reduce the computation speed.

In order to achieve optimum performance by searching for the best delay set without another parameter estimation, in the next section, we derive an optimum scheme based on statistical criteria called joint ML which simultaneously searches for the optimum delay set.

## 3. Proposed Optimum and Sub-Optimum TDE Scheme

In this section, the two proposed TDE schemes are introduced. We first derive the optimum scheme by taking joint ML for TDE. In this section, in order to put forth the mathematical concept simply and easily, the explanations are based on the assumption that the noise signal *z*(*n*) is a random signal and independent identically distributed in trials which follows normal distributions. However, the optimum TDE complexity is quite large, and thus, to reduce complexity, we also propose a sub-optimum TDE, which is a simplified version of the optimum TDE assuming that *x*(*n*) is a random signal but common in each measurement trial. The two schemes only estimate time delay in trials, so that they may avoid the intertwined problem that the other conventional schemes have.

### 3.1. Proposed Optimum TDE Scheme

The proposed TDE scheme, by taking the joint ML, aims to find the most probable point of the time delay vector that consists of the all-trial time delays. The delay vector can be obtained by maximization of the joint pdf of delays as:

(3) $$\begin{array}{cc} \hat{d} & =\underset{\bar{d}}{\arg\;\max}f_{y}(y|\bar{d})\\ & =\underset{\bar{d}}{\arg\;\max}\frac{1}{\sqrt{{\left(2\pi \right)}^{IN}}\left|\Sigma_{\bar{d}}\right|}\exp\left(-\frac{1}{2}{\left(y-\mu_{x}\right)}^{T}\Sigma_{\bar{d}}^{-1}(y-\mu_{x})\right) \end{array}$$

where **y** = [**y**_1_, **y**_2_,…, **y***_I_*]^T^, **y***_i_* = [*y_i_*(1), *y_i_*(2),…, *y_i_*(*N*)], $\bar{d}$ = [${\bar{d}}_{1},{\bar{d}}_{2},\cdots ,{\bar{d}}_{I}$] is a delay vector, **y**^T^ is the transpose of **y**, $\hat{d}$ is the estimated delay vector of $\bar{d}$, and $\Sigma_{\bar{d}}=E\{(y-\mu_{x}){\left(y-\mu_{x}\right)}^{T}\}$ is the covariance matrix which differs according to $\bar{d}$. Note that the previous studies for TDE calculate a cost function for each trial delay, but the cost function of the proposed scheme is based on overall trials, *i.e.*, the delay vector. This means that the proposed scheme focuses on relative delays in trials instead of best delays in each trial. $\Sigma_{\bar{d}}$ is composed of sub-matrices as in:

(4) $$\Sigma_{\bar{d}}=\left(\begin{array}{ccc} r_{\bar{d}}^{1,1} & \cdots & r_{\bar{d}}^{1,I}\\ \vdots & \ddots & \vdots \\ r_{\bar{d}}^{I,1} & \cdots & r_{\bar{d}}^{I,I} \end{array}\right)$$

where $r_{\bar{d}}^{i,j}=E\{(y_{i}-\mu_{x}){\left(y_{i}-\mu_{x}\right)}^{T}\}$ is the covariance sub-matrix for the *i-*th and the *j-th* trial signals. The sub-matrix can be expressed as:

(5) $$r_{\bar{d}}^{i,j}=\left(\begin{array}{ccc} \text{Cov}(y_{i}(1),y_{j}(1)) & \cdots & \text{Cov}(y_{i}(1),y_{j}(N))\\ \vdots & \ddots & \vdots \\ \text{Cov}(y_{i}(N),y_{j}(1)) & \cdots & \text{Cov}(y_{i}(N),y_{j}(N)) \end{array}\right)$$

where Cov(*y_i_*(*n*), *y_j_*(*k*)), which is the covariance of the *n-*th sample in the *i-*th trial and the *k-*th sample in the *j-*th trial, can be determined by the statistical property of *x*(*n*) and (*d_i_* − *d_j_*). This shows that the optimum TDE can be applied just with the statistical information of the ERP signals, and does not require much information about the instantaneous ERP signal, *i.e.*, autocorrelation of the ERP signal instead of ERP signal estimation. Note that the autocovariance function of the ERP signal can be estimated based on the measured signal. For example, the autocovariance functions of each trial signal and their average will provide an estimated autocorrelation. Since the autocorrelation and autocovariance function are based on how fast the ERP changes, they are easier to estimate than the signals themselves which need to obtain the template signal in the conventional schemes. In addition, the time delay vector $\bar{d}$ contains the delays of all the trials. The joint ML cost function in Equation (3) shows that it is maximum where the overall time delays are well estimated, resulting in better performance than other conventional methods employing estimation of individual trials like Equation (2).

### 3.2. Proposed Sub-Optimum TDE Scheme

The optimum scheme performs the best from the joint ML point of view. However, it involves great complexity because of the calculation of $\Sigma_{\bar{d}}^{-1}$ and $\left|\Sigma_{\bar{d}}\right|$ in Equation (3). Hence, we propose a sub-optimal TDE with a lower computational burden assuming that *x*(*n*) is a random signal but common in each measurement trials. 

The exponential term in Equation (3) plays a key role for the joint ML calculation. Taking logarithm can make the Equation (3) approximately simplified as:

(6) $$\hat{d}\approx \underset{\bar{d}}{\arg\;\max}\left[-\frac{1}{2}{\left(y-\mu_{x}\right)}^{T}\Sigma_{\bar{d}}^{-1}(y-\mu_{x})\right]$$

For further simplification, $\Sigma_{\bar{d}}^{-1}$ is simplified by taking only major elements in the matrix. The following example explains the simplification.

For intuitive understanding of the covariance matrix simplification, we consider a simple case where *x*(*n*) is a white Gaussian random signal with variance $\sigma_{x}^{2}$, and noise *z_i_*(*n*) is zero mean white Gaussian noise with variance $\sigma_{z}^{2}$. In the case of the white random signal *x*(*n*), $r_{\bar{d}}^{i,j}$ has a non-zero value at the upper or lower diagonal elements determined by (*d_i_* − *d_j_*). The main diagonal elements have non-zero value $\left(\sigma_{x}^{2}+\sigma_{z}^{2}\right)$ for *i* = *j*, and the shifted sub-diagonal elements have $\sigma_{x}^{2}$ for *i ≠ j*. The non-zero elements in the shifted diagonal represent the relative delay between the *i*-th and the *j*-th trial. For example, when there are three trial data sequences each three points long for each trial (*I* = *N* = 3), $\sigma_{x}^{2}$ = 1, $\sigma_{z}^{2}$ = 0.1, the covariance matrix of a delay set $\bar{d}$ = [0, 0, 1] is a 9 × 9 matrix and represented as:

(7) $$\Sigma_{\bar{d}}=\left[\begin{array}{ccc} \begin{array}{ccc} 1.1 & 0 & 0\\ 0 & 1.1 & 0\\ 0 & 0 & 1.1 \end{array} & \begin{array}{ccc} 1 & 0 & 0\\ 0 & 1 & 0\\ 0 & 0 & 1 \end{array} & \begin{array}{ccc} 0 & 1 & 0\\ 0 & 0 & 1\\ 0 & 0 & 0 \end{array}\\ \begin{array}{ccc} 1 & 0 & 0\\ 0 & 1 & 0\\ 0 & 0 & 1 \end{array} & \begin{array}{ccc} 1.1 & 0 & 0\\ 0 & 1.1 & 0\\ 0 & 0 & 1.1 \end{array} & \begin{array}{ccc} 0 & 1 & 0\\ 0 & 0 & 1\\ 0 & 0 & 0 \end{array}\\ \begin{array}{ccc} 0 & 0 & 0\\ 1 & 0 & 0\\ 0 & 1 & 0 \end{array} & \begin{array}{ccc} 0 & 0 & 0\\ 1 & 0 & 0\\ 0 & 1 & 0 \end{array} & \begin{array}{ccc} 1.1 & 0 & 0\\ 0 & 1.1 & 0\\ 0 & 0 & 1.1 \end{array} \end{array}\right]$$

The main diagonal elements in Equation (7) have $\left(\sigma_{x}^{2}+\sigma_{z}^{2}\right)$ = 1.1, and the sub-matrix $r_{\bar{d}}^{i,j}$ where *i ≠ j* has a non-zero element according to the delay set. For example:

(8) $$r_{\bar{d}}^{1,2}={\left(r_{\bar{d}}^{2,1}\right)}^{T}=\left[\begin{array}{ccc} 1 & 0 & 0\\ 0 & 1 & 0\\ 0 & 0 & 1 \end{array}\right]$$

and this matrix has $\sigma_{x}^{2}$ = 1 in the diagonal elements since the 1st and 2nd trial delays in $\bar{d}$ are the same. However, the non-zero terms in $r_{\bar{d}}^{1,3}$ are shifted from the main diagonal entries due to the delay difference (*d_i_* − *d_j_*), *i.e.*, they are shifted by 1 with (*d*_1_ − *d_3_*) = −1 as:

(9) $$r_{\bar{d}}^{1,3}={\left(r_{\bar{d}}^{3,1}\right)}^{T}=\left[\begin{array}{ccc} 0 & 1 & 0\\ 0 & 0 & 1\\ 0 & 0 & 0 \end{array}\right]$$

For all the sub-matrices, the element in the *a-*th row and *b-*th column, of $r_{\bar{d}}^{i,j}$ can be expressed as:

(10) $$r_{\bar{d}}^{i,j}(a,b)=\sigma_{x}^{2}\delta (a+{\bar{d}}_{i}-b-{\bar{d}}_{j})+\sigma_{z}^{2}\delta (a-b)\delta (i-j)$$

where δ(n) is the Dirac delta function. Then $\Sigma_{\bar{d}}^{-1}$ is presented as:

(11) $$\Sigma_{\bar{d}}^{-1}=\left[\begin{array}{ccc} \begin{array}{ccc} 6.8 & 0 & 0\\ 0 & 6.8 & 0\\ 0 & 0 & 5.2 \end{array} & \begin{array}{ccc} -3.2 & 0 & 0\\ 0 & -3.2 & 0\\ 0 & 0 & -4.8 \end{array} & \begin{array}{ccc} 0 & -3.2 & 0\\ 0 & 0 & -3.2\\ 0 & 0 & 0 \end{array}\\ \begin{array}{ccc} -3.2 & 0 & 0\\ 0 & -3.2 & 0\\ 0 & 0 & -4.8 \end{array} & \begin{array}{ccc} 6.8 & 0 & 0\\ 0 & 6.8 & 0\\ 0 & 0 & 5.2 \end{array} & \begin{array}{ccc} 0 & -3.2 & 0\\ 0 & 0 & -3.2\\ 0 & 0 & 0 \end{array}\\ \begin{array}{ccc} 0 & 0 & 0\\ -3.2 & 0 & 0\\ 0 & -3.2 & 0 \end{array} & \begin{array}{ccc} 0 & 0 & 0\\ -3.2 & 0 & 0\\ 0 & -3.2 & 0 \end{array} & \begin{array}{ccc} 0.9 & 0 & 0\\ 0 & 6.8 & 0\\ 0 & 0 & 6.8 \end{array} \end{array}\right]$$

From Equation (11), we observe that $\Sigma_{\bar{d}}^{-1}$ has non-zero elements at the same position as $\Sigma_{\bar{d}}$ has. As Equation (11) shows, $\Sigma_{\bar{d}}^{-1}$ preserves the location of the sub-diagonal shift using the delay set, and this determines how (**y** − *μ_x_*)*^T^*, $\Sigma_{\bar{d}}^{-1}$, and (**y** − *μ_x_*) are multiplied. For example, the metric in Equation (6) can be obtained by taking the approximation of $\Sigma_{\bar{d}}^{-1}$ as:

(12) $$\Sigma_{\bar{d}}^{-1}\approx \left[\begin{array}{ccc} \begin{array}{ccc} 1 & 0 & 0\\ 0 & 1 & 0\\ 0 & 0 & 1 \end{array} & \begin{array}{ccc} -1 & 0 & 0\\ 0 & -1 & 0\\ 0 & 0 & -1 \end{array} & \begin{array}{ccc} 0 & -1 & 0\\ 0 & 0 & -1\\ 0 & 0 & 0 \end{array}\\ \begin{array}{ccc} -1 & 0 & 0\\ 0 & -1 & 0\\ 0 & 0 & -1 \end{array} & \begin{array}{ccc} 1 & 0 & 0\\ 0 & 1 & 0\\ 0 & 0 & 1 \end{array} & \begin{array}{ccc} 0 & -1 & 0\\ 0 & 0 & -1\\ 0 & 0 & 0 \end{array}\\ \begin{array}{ccc} 0 & 0 & 0\\ -1 & 0 & 0\\ 0 & -1 & 0 \end{array} & \begin{array}{ccc} 0 & 0 & 0\\ -1 & 0 & 0\\ 0 & -1 & 0 \end{array} & \begin{array}{ccc} 1 & 0 & 0\\ 0 & 1 & 0\\ 0 & 0 & 1 \end{array} \end{array}\right]$$

The zero value elements in $\Sigma_{\bar{d}}^{-1}$ do not need to be calculated since the multiplication result is zero, enabling computation complexity reduction. Furthermore, if we set the non-zero elements in $\Sigma_{\bar{d}}^{-1}$ at a constant number (*i.e.*, −1 as in Equation (12)), multiplication is required only for the corresponding delay values for Equation (6). In addition, the main diagonal in Equation (12) can be disregarded in the computation of Equation (6) since it is common irrespective of $\bar{d}$. Finally, the sub-optimum scheme can be represented as:

(13) $$\hat{d}\approx \underset{\bar{d}}{\arg\;\max}\left[\sum_{j}\sum_{i\neq j}\sum_{n}\left(y_{i}(n)-\mu_{y})(y_{j}(n-(d_{j}-d_{i}))-\mu_{y}\right)\right]$$

In the sub-optimal scheme, as shown in Equation (14), $\hat{d}$ is obtained using only the correlation of the measured signal **y**. An exhaustive search or a stochastic optimization can be applied for searching $\hat{d}$. Hence the optimum and sub-optimum joint ML solutions choose the best relative trial delay vector satisfying Equation (3) and a close to the best relative trial delay vector, respectively, from a ML point of view, among the relative delay vectors of all the trials.

**Figure 1:**
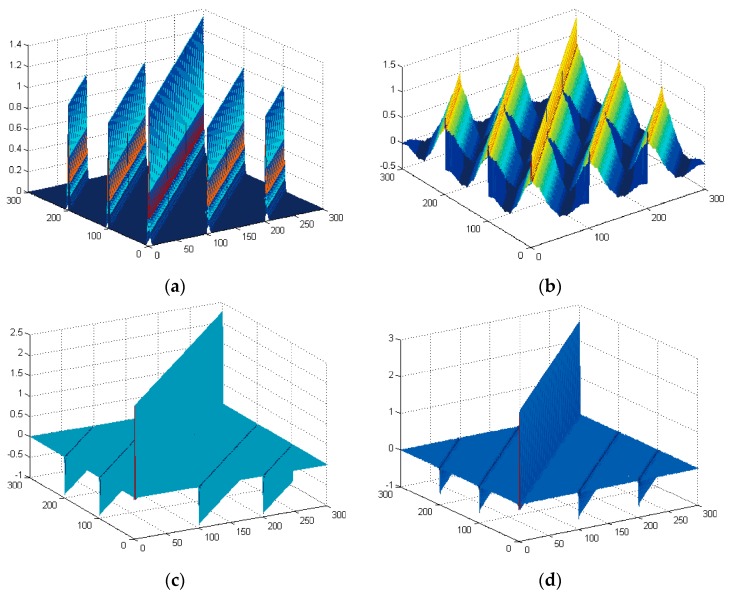
(**a**,**b**) show the covariance matrix of colored random signal and real EEG signal respectively; and (**c**,**d**) are their inverse covariance matrix, respectively.

In Equation (11), the values of the non-zero elements are different, e.g., 6.8 ≠ 5.2 ≠ 0.9 and –3.2 ≠ –4.8, but as the matrix size increases, with larger *N* and *I*, this difference decreases, resulting in smaller variance among the non-zero elements. Figure 1 shows 3D color display of covariance matrix $\Sigma_{\bar{d}}$ and inverse covariance matrix $\Sigma_{\bar{d}}^{-1}$ of signals of *N* = 100 and *I* = 3. Figure 1a,b show $\Sigma_{\bar{d}}$ of a colored random signal and a real EEG signal, respectively, and Figure 1c,d are their $\Sigma_{\bar{d}}^{-1}$, respectively. As seen in Figure 1c, the main diagonal and sub-diagonal values are similar, meaning that $\Sigma_{\bar{d}}^{-1}$ can be approximated to have constant non-zero terms as in Equation (12). A similar tendency can be observed for real EEG signals which are not white random signals as seen in Figure 1b,d. This implies that the sub-optimal scheme can be applied for EEG signals that contain ERP signals although the scheme is derived based on a white random signal assumption. In addition, if *x*(*n*) is a white random signal, Equations (3) and (13) will return almost the same result. On the other hand, the performance of the sub-optimal scheme will be worse than that of the optimum scheme if the ERP signal does not follow white random signal characteristics due to the larger approximation error of $\Sigma_{\bar{d}}^{-1}$. Since the sub-optimum scheme does not require complicated operations such as matrix inversion, the computational complexity is reduced dramatically. The computation complexity of the sub-optimum scheme is O(*R^I^I*^2^*R*), which is far smaller than that of the optimum scheme, which is O(*R^I^*(*IR*)^2.373^), where *R* (*R* ≤ *N*) is the searching range of the delay candidates. Hence, for faster execution, the sub-optimal scheme can be applied.

## 4. Performance Evaluation

### 4.1. Simulation Setup

#### 4.1.1. Signal Selection

We have derived the proposed TDE schemes by taking the joint ML criterion, considering the ERP signal as a Gaussian random signal. The sub-optimal scheme is obtained by approximation of the optimum scheme, so that there may be performance degradation due to this approximation. In order to evaluate the performance of the proposed schemes, we first compare the performance of the optimal and the sub-optimal schemes for white (w1) and colored (w2) random signals. Then, in order to evaluate the performance with more realistic ERP signals, deterministic signal and EEG signal simulation are also presented.

**Figure 2:**
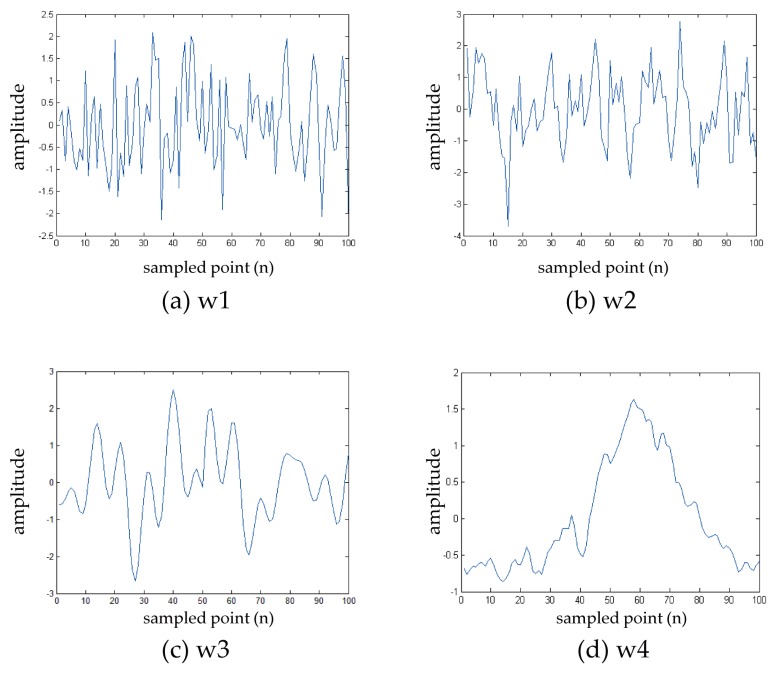
Signal waveforms for simulations. (**a**) White random signal; (**b**) colored random signal; (**c**) deterministic signal; and (**d**) EEG signal.

The colored random signal is generated using autoregressive (AR) modeling as:

(14) $$x(n)=\alpha x(n-1)+\sqrt{1-\alpha^{2}}w(n)$$

where *w*(*n*) is a random signal that follows time independent Gaussian distribution. We set *α* = 0 for the white random signal (w1) and *α* = 0.5 for the colored signal (w2). Second, we test the performance of the proposed schemes using a deterministic signal (w3). The deterministic signal is generated by combining multiple sinusoidal waves with frequencies chosen randomly from range 4–16 Hz [26]. Finally, we apply a EEG signal (w4) simulation that uses a measured real EEG signal which is a five-box task result [27] to confirm how the schemes perform on real data. Figure 2 shows the four signal waveform shapes used for random (w1, w2), deterministic (w3), and EEG signal simulation (w4). The real ERP signal data that is measured by EEG recordings includes an inherent noise term whose power is predetermined. In order to obtain performance results with different SNR, we apply the EEG simulation that was introduced in [25,28,29], where the SNR is made to be controllable, and the signal morphology and noise structure are preserved close to those of the real ERP signal [25]. The EEG signal simulation averages *I* trial signals to obtain ERP signal $\overset{⌢}{x}(n)$ as:

(15) $$\overset{⌢}{x}(n)=\frac{1}{I}\sum_{i}^{I}y_{i}(n)$$

and the residual signal is regarded as the noise ${\overset{⌢}{n}}_{i}(n)$ as:

(16) $${\overset{⌢}{n}}_{i}(n)=y_{i}(n)-\overset{⌢}{x}(n)$$

The reconstructed *i-*th trial data ${\overset{⌢}{y}}_{i}(n)$ is composed of $\overset{⌢}{x}(n)$ with different delay and ${\overset{⌢}{n}}_{i}(n)$ as:

(17) $${\overset{⌢}{y}}_{i}(n)=\phi {\overset{⌢}{n}}_{i}(n)+\overset{⌢}{x}(n-d_{i})$$

where ϕ is a control factor determining the SNR. We fix the signal power and adjust the noise power by controlling ϕ to obtain different SNR as in [25]. Note that $\overset{⌢}{x}(n)$ is the common ERP signal, which is w4 signal in Figure 2d regarded to have no time delay error, and the time delay of ${\overset{⌢}{y}}_{i}(n)$ in each trial is controlled by *d_i_* as in Equation (17). In EEG signal simulation based on Equation (17), we use the EEG data set obtained by Makeig *et al.* [27] for 15 health controls. The EEG is recorded while the subjects are doing a five-box task. In the experiment, there are five empty boxes in a monitor, and one of the boxes is selected as the target box. During the experiment, subjects are asked to watch the target box and press a button as soon as possible when the target box is filled with a disk. The disk is displayed for 117 ms and the disk appears on the five empty boxes in a random manner with inter-stimulus intervals of 250 to 1000 ms. The EEG recordings are collected from 29 scalp electrodes based on a modified international 10–20 system. The data is sampled at 128 Hz. For simulation performance evaluation, five sets of trial data (*i.e*., *y_i_*(*n*), *i* = 1, 2,…, *I* = 5) are used, and for more practical analysis in ERP analysis part, 100 trial data (*i.e*., *y_i_*(*n*), *i* = 1, 2,…, *I* = 100) located at Pz are chosen from the task when the subjects respond to the target. The trials are filtered with a 50 Hz low pass filter.

#### 4.1.2. TDE Performance Evaluation

Two different evaluation methods are utilized for the TDE algorithm performance evaluation. The first method is the expected time delay error estimation with respect to SNR. The four signals, two random signals (w1 and w2), deterministic signal (w3), and the EEG signal (w4) are used for this performance evaluation. In this simulation, white Gaussian noise is added to the (w1–w3) signals at different power levels according to SNR and the signal length is 100 points long (N=100). For EEG signal (w4) simulation, ϕ is controlled for different level of SNR. For each signal, five trials with a random delay *d_i_* following a uniform distribution in [–5, 5] are used as Equation (17), and the four schemes are applied to estimate *d_i_* . In order to evaluate the performance statistically, the simulation is repeated 1000 times. We set the search range as much as the random delay range. The performance is evaluated in terms of the expected of root-mean-square error (RMSE) of the estimated time delay ${\hat{d}}_{i}$ as:

(18) $$\lambda_{est}=E\left(\sqrt{\frac{1}{I}\sum_{i=1}^{I}{\left({\hat{d}}_{i}-d_{i}\right)}^{2}}\right)$$

The second method is ERP analysis such as peak amplitude and peak latency, and spectral perturbation analysis. An artificial random delay *d_i_* following a uniform distribution in [−200 ms, +200 ms] are applied to the EEG signals from 15 subjects. In order to observe the effect of time delay realignment on the ERP waveform, realigning is applied as a preprocessing step to the EEG signals. There are six different ERP analysis results when obtaining the realigned and averaged signal $\overset{⌣}{y}(n)=\frac{1}{I}\sum_{i=1}^{I}{\overset{⌢}{y}}_{i}(n+{\hat{d}}_{i}^{})$ averaged ERP result of the original ERP signal without timing errors (*i.e.*, perfectly aligned ERP corresponding to *d_i_* = 0 in Equation (17)), the ERP signal without realignment (*i.e.*, ${\hat{d}}_{i}$ = 0(≠ *d_i_*) without timing correction), and realigned ERP by the four different algorithms (*i.e.*, ${\hat{d}}_{i}$ is different according to the applied algorithms). After TDE realignment and averaging, the maximum positive peak after 300 ms from the stimulus onset (P3) and its time delay are calculated as the peak amplitude and peak latency, respectively. In order to explain the ERP analysis statistically, ERP analysis of 15 subjects is performed and their mean and standard deviation (SD) are calculated. Paired T-test is applied to evaluate the performance difference between the proposed schemes and the conventional schemes. The spectral perturbation is also performed after TDE realignment. Time-frequency domain perturbation data is calculated using the EEGLAB toolbox [30] based on a short-time Fourier transform (STFT) with window size of 0.5 s, sliding step of 15 ms, and the perturbation color maps are limited by a −10 dB to +10 dB scale. Thus, the time-frequency domain data is averaged in trials for spectral perturbation.

### 4.2. Simulation Result

#### 4.2.1. Random Signal

**Figure 3:**
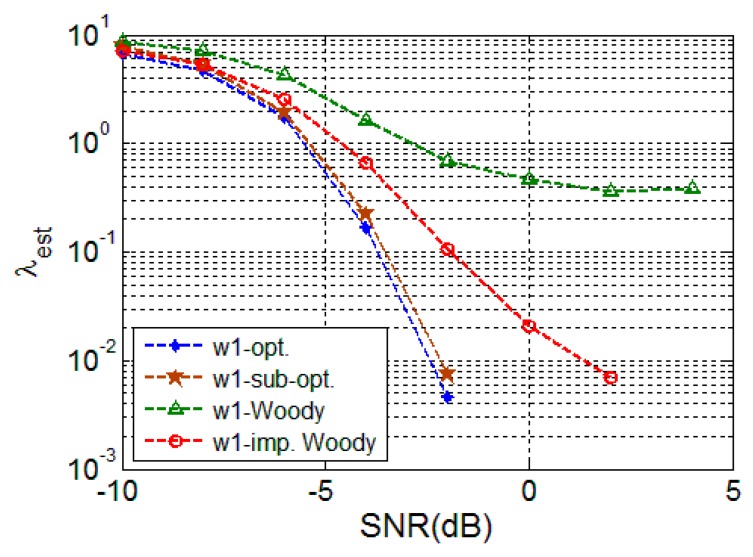
TDE performance for white random signal (w1).

**Figure 4:**
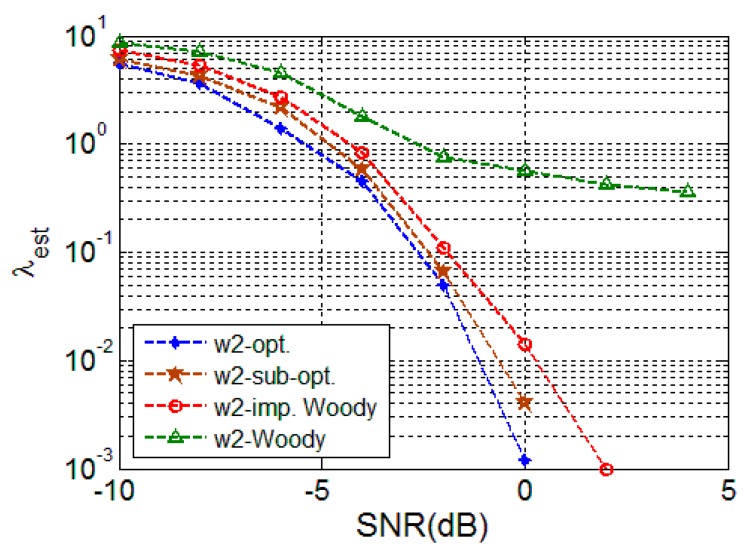
TDE performance for colored random signal (w2).

Two types of random signals are used for this random signal simulation. One of the random signal simulations uses a white random signal (w1). In addition, a colored random signal is used to analyze the performance difference from white random signal performance. Figure 3 and Figure 4 plot *λ_est_* of the conventional and proposed schemes when the random signals are employed. Woody [19], and improved Woody [23] schemes are compared as conventional schemes.

These figures show that the optimum TDE performs the best, followed by the sub-optimal scheme; the proposed schemes provide significant gain over the conventional schemes, especially for the white random signal case (w1). Note that the Woody scheme experiences saturation over a certain SNR. This is because the signal estimation and the time delay estimation are intertwined. Unless time delay errors in different trials are corrected simultaneously, the template cannot avoid the intertwining problem. Therefore, there is higher possibility that the single delay estimation will have an erroneous template. The improved Woody scheme can reduce the performance degradation, but it is still much worse than those of the proposed schemes.

As shown in Figure 4, a similar tendency can be observed except that the performance gap between the proposed optimal and suboptimal schemes is larger for the colored random signal than it is for the white random signal since the covariance matrix pattern is more different from the approximation form of the covariance matrix Equation (12). However, the gains with the use of the proposed schemes are still large, implying their benefit in use.

#### 4.2.2. Deterministic Signal

**Figure 5:**
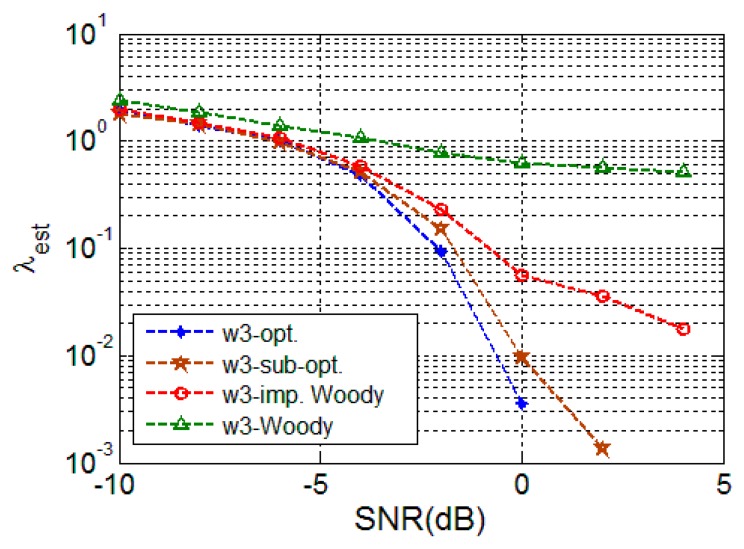
TDE performance for deterministic signal (w3).

Figure 5 depicts the performance of the deterministic signals where a similar tendency toward random signals can be observed. This implies that the proposed schemes, derived with the assumption of random signals, can still provide a large performance gain even for deterministic signals since they are based on maximization of autocorrelation of the signal of all the candidate delays.

#### 4.2.3. EEG Signal

**Figure 6:**
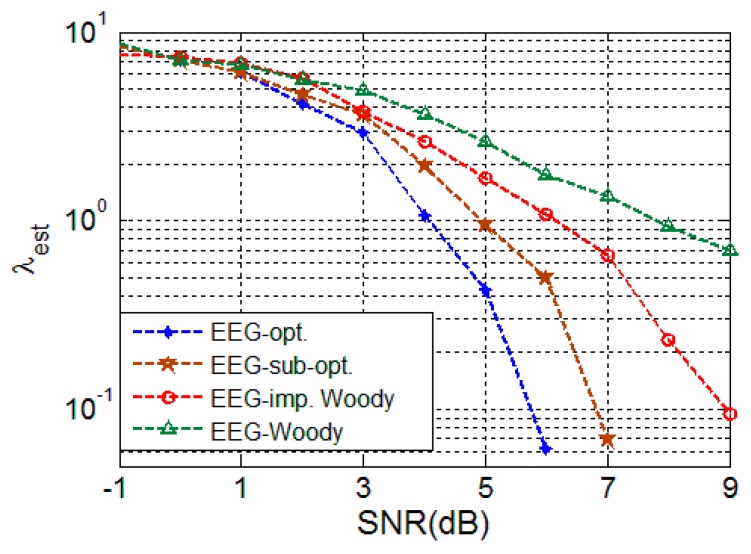
TDE performance for EEG signal (w4).

Figure 6 shows that the two proposed schemes outperform the conventional schemes. The proposed schemes provide the same performance of expected time error with less SNR. The expected delay error performance *λ_est_* = 10^0^ is achieved with SNR = 4 dB and 5 dB for the proposed optimal and the sub-optimal schemes, respectively, while the conventional schemes achieve with SNR = 6 dB and 8 dB for the improved Woody and Woody methods, respectively. The Woody scheme simulation results indicates a severe case of an intertwining problem, which causes saturation; the improved Woody scheme performance shows partial performance degradation due to the intertwining problem. The performance of the two proposed schemes is better since they can avoid the intertwining problem due to the fact they do not need reference signal estimation.

While the proposed schemes provide better performance, the proposed schemes have higher computational complexity. However, this computation problem is less crucial for off-line signal processing and will be less demanding as computation capability improves with hardware system advances. Hence, the proposed scheme can be a reasonable solution for better TDE performance.

#### 4.2.4. ERP Analysis

**Figure 7:**
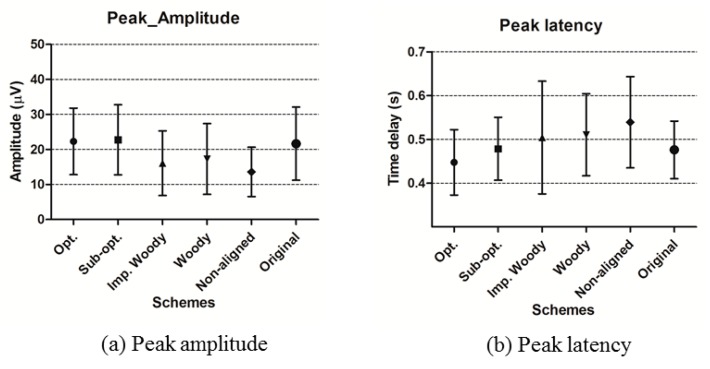
Statistical analysis of ERP characteristic in time domain. (**a**) Peak amplitude; (**b**) Peak latency analysis result with 15 subjects.

It can be expected that as the time delay error increases, ERP characteristics will differ in time and frequency domain. In order to observe the time delay error effect and the compensated result by TDE schemes, we perform ERP characteristic analysis in the time domain and spectral perturbation for 15 subject data. Figure 7 shows a statistical analysis of P3 peak amplitude and latency. In the peak amplitude result, the proposed schemes have less than 2 μV mean error while the result in the conventional schemes have larger than 4 μV mean error. In addition, there is less than 30 ms time latency error between the proposed schemes and the original signal, while the conventional and non-aligned signal have more than 40 ms latency error with larger SD. The performance difference between the proposed schemes and conventional schemes in peak amplitude and latency analysis are significant (*p* < 0.05). Note that the proposed two schemes have less difference in the mean amplitude as well as SD to the original than that of improved Woody and Woody schemes, and non-aligned ERP. Therefore, the proposed TDE schemes can correct time delays more accurately than conventional schemes.

**Figure 8:**
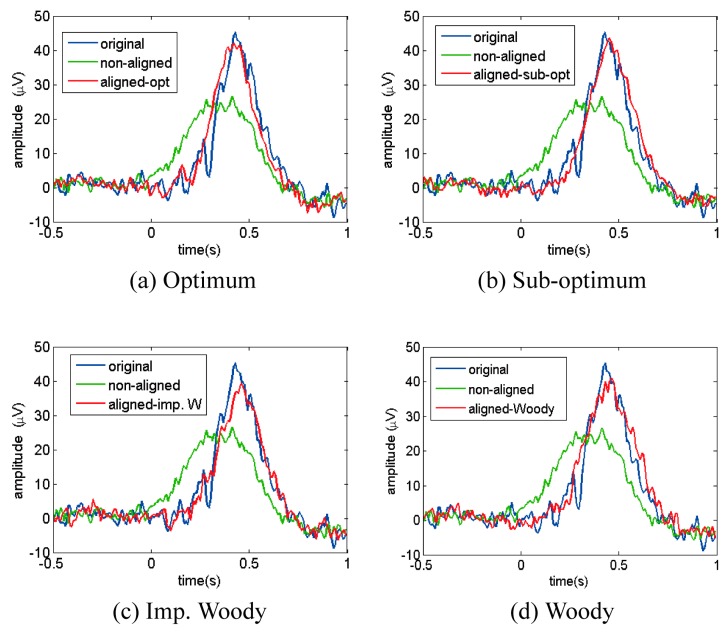
Averaged time domain ERP signal comparison between original ERP (blue dot line), ERP without realignment (green dot and dash line), and realigned signal by applying the (**a**) optimal; (**b**) sub-optimal; (**c**) improved Woody; and (**d**) Woody scheme (red solid line).

Figure 8 shows an example of ERP analysis in time domain. It shows that the non-aligned ERP signal that experiences random time delay error has the lowest P3 peak amplitude, and realigned ERP signal compensated by the improved Woody and Woody schemes in Figure 8c,d have smaller peak amplitudes than the original ERP signal, while the realigned signal shapes with the use of the proposed schemes in Figure 8a,b are more similar to the original ERP.

**Figure 9:**
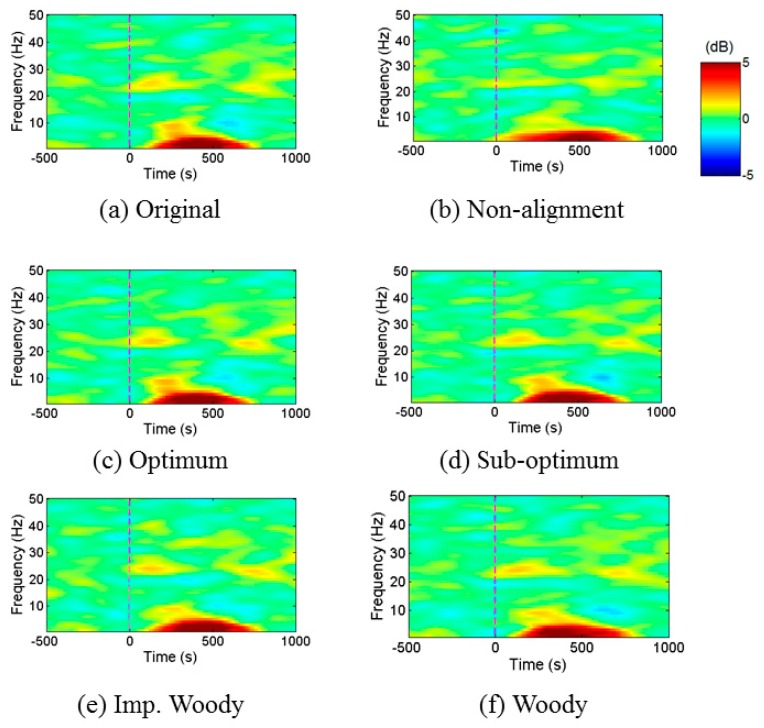
Event related spectral perturbation analysis with (**a**) Original ERP; (**b**) ERP without realignment; and ERP after realignment by applying the (**c**) optimal; (**d**) sub-optimal; (**e**) improved Woody; and (**f**) Woody schemes.

Figure 9 shows a spectral perturbation result of the original ERP signal without timing errors, the ERP signal without realignment, and realigned ERP by the four different schemes. All the results, except non-aligned signal result, show similar synchronized activation on the delta (0–4 Hz), theta (4–7 Hz) and beta bands (13–30 Hz) shown in red and yellow color. However, the result of conventional schemes shows weaker activation on the alpha band (7–13 Hz) compared to the proposed schemes. Note that the TDE schemes provide much more similar results than the non-alignment result that shows only blurred activation on the delta and theta bands. This means that we can obtain better spectral perturbation images with the use of delay realignment employing the proposed TDE schemes.

## 5. Conclusions

TDE is an effective technique for bio-signal analysis, especially for repetitive and evoked electrical signals such as ERP. We have proposed a joint ML-based optimal and sub-optimal TDE that do not require estimation of an ERP reference signal and estimate the delay vector in a joint manner. According to the simulation results, compared with those of the conventional schemes, our proposed optimal scheme can correct time errors much more accurately at a given SNR or can achieve much less SNR, e.g., up to 4 dB at *λ_est_* = 10°, for the same performance of the expected time error, while it needs a large number of computations. The proposed sub-optimal schemes can reduce this complexity burden significantly at the cost of small performance degradation. Thus the proposed scheme can be very effective when the estimation performance is critical, especially when the reference signal is unknown or random.
